# Virtual Boot Camp: COVID‐19 evolution and structural biology

**DOI:** 10.1002/bmb.21428

**Published:** 2020-08-14

**Authors:** Stephen K. Burley, Yana Bromberg, Paul Craig, Siobain Duffy, Shuchismita Dutta, Bonnie L. Hall, Brian P. Hudson, Jennifer Jiang, Sagar D Khare, Julia R. Koeppe, Joseph H. Lubin, Stephen A. Mills, Michael J. Pikaart, Rebecca Roberts, Vidur Sarma, Jitendra Singh, Jay A. Tischfield, Lingjun Xie, Christine Zardecki

**Affiliations:** ^1^ Research Collaboratory for Structural Bioinformatics Protein Data Bank, Rutgers The State University of New Jersey Piscataway New Jersey USA; ^2^ Institute for Quantitative Biomedicine, Rutgers The State University of New Jersey Piscataway New Jersey USA; ^3^ Research Collaboratory for Structural Bioinformatics Protein Data Bank, San Diego Supercomputer Center University of California, San Diego La Jolla California USA; ^4^ Rutgers Cancer Institute of New Jersey, Rutgers The State University of New Jersey New Brunswick New Jersey USA; ^5^ Department of Biochemistry and Microbiology Rutgers University New Brunswick New Jersey USA; ^6^ School of Chemistry & Materials Science Rochester Institute of Technology Rochester New York USA; ^7^ Department of Ecology, Evolution and Natural Resources, School of Environmental and Biological Sciences, Rutgers The State University of New Jersey New Brunswick New Jersey USA; ^8^ Department of Chemistry Grand View University Des Moines Iowa USA; ^9^ Department of Chemistry & Chemical Biology Rutgers University Piscataway New Jersey USA; ^10^ Chemistry State University of New York at Oswego Oswego New York USA; ^11^ Chemistry Department Xavier University Cincinnati Ohio USA; ^12^ Department of Chemistry and Biochemistry Hope College Holland Michigan USA; ^13^ Biology Department Ursinus College Collegeville Pennsylvania USA; ^14^ CUNY New York City College of Technology New York New York USA; ^15^ Department of Genetics Rutgers University Piscataway New Jersey USA; ^16^ Human Genetics Institute of New Jersey Piscataway New Jersey USA

COVID‐19‐related campus closures necessitated invention of an intensive online learning experience that introduced students to Structural Bioinformatics. The program engaged 33 students from 11 different institutions ranging from high school to graduate school. The objectives of the intensive, 1‐week Boot Camp focused on helping students build a foundation for studying the SARS‐CoV‐2 Main Protease (Nsp5) to understand how the protein evolved during the first 6 months of the COVID‐19 pandemic by exploring amino acid *sequence* and 3D atomic‐level *structure* using various structural bioinformatics tools. Participants analyzed how the protein changed as the virus spread around the world, comparing Nsp5 from the original viral isolate to 161 unique sequence/structure variants.

Hosted by the RCSB Protein Data Bank (RCSB.org)^1^, ^2^ and the Rutgers University Institute for Quantitative Biomedicine (IQB; iqb.rutgers.edu), the course met daily for 5 days, utilizing local expertise to present topics related to COVID‐19 function and evolution (e.g., evolution of RNA viruses), introductions to tools (e.g., Clustal Omega^3^ for sequence alignments and phylogenetic trees; Mol*^4^ for 3D molecular visualization; and Foldit^5^ for structural/energetic effects of sequence mutations), and related team activities. The Boot Camp also included lectures about the COVID‐19 pandemic, SARS‐CoV‐2 protein structures (notably Nsp5 and the Spike protein), and the Rutgers RUCDR saliva test for detecting COVID‐19. After 5 days in close collaboration, 10 student/mentor teams presented their findings on the energetic effects of the 161 mutations on the plasticity of the Nsp5 3D structure.

The camp was designed and directed by members from the IQB and the RCSB Protein Data Bank, facilitated by nine mentors from Rutgers and six mentors associated with the BASIL Consortium (https://basilbiochem.github.io/basil/index.html ^6^). Student researchers were selected from the RISE program at Rutgers (https://www.rise.rutgers.edu/), from other Rutgers‐affiliated programs, and from the campuses of the BASIL mentors. Most students were Chemistry/Biochemistry majors without prior programming experience.

Daily sessions ran approximately 6 h, including breaks and lunch. Most active learning took place in virtual breakout rooms where teams worked through data analysis exercises with their mentors. Progress vs. goals was reviewed during 30‐min faculty/mentor meetings at the close of each day.

The major challenge in delivering an online experience was monitoring student engagement. Zoom video conferencing breakout rooms were key to providing space for students and mentors to interact with the material in small groups. A parallel Slack communication channel was also helpful in communicating across groups. Other lessons learned included the need for enforced break times and abbreviated lecture formats.

Overall student learning will be assessed by the faculty and mentors using student deliverables (reports and presentations) and an exit survey. In addition to an increased understanding of COVID‐19, initial survey responses indicate that most participants felt they had increased their computational proficiency (FoldIt) and their knowledge of structural biology (protein secondary structure, molecular energetics, molecular modeling, and the Protein Data Bank).

Select materials for replicating Boot Camp modules are available online for reuse at https://covid19-bootcamp.rcsb.org.
